# Setting Global Research Priorities in Pediatric and Adolescent HIV Using the Child Health and Nutrition Research Initiative (CHNRI) Methodology

**DOI:** 10.1097/QAI.0000000000001742

**Published:** 2018-07-11

**Authors:** Cadi Irvine, Alice Armstrong, Jason M. Nagata, Nigel Rollins, Diddie Schaaf, Meg Doherty, Martina Penazzato, Marissa Vicari

**Affiliations:** * HIV Programmes and Advocacy, International AIDS Society, Geneva, Switzerland;; † Department of HIV and Global Hepatitis Programme, World Health Organization, Geneva, Switzerland;; ‡ Division of Adolescent and Young Adult Medicine, Department of Pediatrics, University of California, San Francisco, CA; and; § Department of Maternal, Newborn, Child and Adolescent Health, World Health Organization, Geneva, Switzerland.

**Keywords:** Child Health and Nutrition Research Initiative (CHNRI), research priorities, adolescent HIV, pediatric HIV, AIDS, research agenda

## Abstract

**Background:**

WHO and the Collaborative Initiative for Paediatric HIV Education and Research (CIPHER) of the International AIDS Society (IAS) led a collaborative process to set global prioritized research agendas, aiming to focusing future research, funding, and stakeholder's efforts. This study describes the methodology used to establish the research agendas.

**Methods:**

The Child Health and Nutrition Research Initiative methodology was adapted in parallel exercises on pediatric and adolescent HIV. After definition of scope by an expert working group, priority questions were collected from stakeholders through an online survey. Submitted questions were coded, analyzed, and collated. The same respondents were asked to score the collated lists through a second online survey. The top 10 ranked questions per thematic area (testing, treatment, and service delivery) were reviewed and priority themes developed with consideration of existing policy, systematic reviews, and planned, ongoing, and recently published research.

**Results:**

A total of 375 respondents submitted 1735 priority research questions. The majority of respondents were from Africa; 55% self-identified as researchers. The final collated lists included 51 and 61 research questions for pediatric and adolescent HIV, respectively. The response rate for the second survey was 48%. The final research agendas include 5 priority research themes per area, discussed in 2 separate publications.

**Conclusions:**

To date, this is the largest example of the Child Health and Nutrition Research Initiative method in pediatric and adolescent HIV in terms of stakeholders reached, and the first to incorporate top thematic areas based on current evidence. Its impact on improving outcomes for these populations will require strong political and financial commitment.

## INTRODUCTION

To date, despite great advances in prevention, testing, and treatment, HIV in children and adolescents remains a major cause of mortality and morbidity in these vulnerable populations.^1^ The main body of HIV research typically addresses biomedical and service delivery questions in the adult population. These findings are not always generalizable to younger populations due to differences in the natural history of the disease as well as their unique needs. Evidence is needed on how to successfully address their specific health needs including how best to deliver services to them.^2^ This is critical to inform policy and reach the ambitious targets that the global community has set to achieve an AIDS-free generation by 2030.^3^

Setting priorities for health research is a complex process and, over the past decade, there has been an increase in the development and use of various new methods. A review of approaches used to set health research priorities identified the Child Health and Nutrition Research Initiative (CHNRI) method as the approach most commonly implemented.^4^ Over the last decade, CHNRI has become a leading prioritization methodology with over 50 published examples in the literature.^4,5^ Thorough guidance is available on its use while still providing the flexibility to adapt to different objectives and contexts. The aim of CHNRI is to prioritize health research questions that, if answered, would reduce disease burden and inequities in populations.^6^ CHNRI also allows for broad stakeholder involvement, facilitating better reflection of the priorities across the health response, including on the ground, and shared ownership of the research agenda. In addition, it has been widely endorsed by the World Health Organization (WHO) in several research prioritization exercises^5,7–9^ as part of its mandate to shape global research agendas and stimulate the generation, translation, and dissemination of valuable knowledge.

Competing priorities and rapidly diminishing resources for the HIV response^10^ require that efforts are responsive to gaps and needs and streamlined to maximize the impact of future research investments in HIV for children and adolescents. Toward this, a collaborative process was undertaken by WHO and the Collaborative Initiative for Paediatric HIV Education and Research (CIPHER) of the International AIDS Society (IAS) to establish global research agendas in pediatric and adolescent HIV. The aim is for all stakeholders in the research process, including the end users of the research, to take ownership of the agendas and promote them through their respective roles. The methods outlined in this article describe the adapted CHNRI approach used to establish the research agendas. The results and greater context of the process are provided in companion papers.^11,12^

## METHODS

To set research priorities in pediatric and adolescent HIV, the CHNRI methodology^7,13^ was followed with an additional phase taking into consideration the current context and evolving HIV research landscape. The 5 phases, described below, were performed to establish a list of priority research questions and final themes.

### Phase 1: Establishment of Expert Working Group and Defining the Scope of the Exercise

An overarching expert working group of 19 members, comprising of pediatric and adolescent HIV experts, were identified by the WHO and CIPHER coordinating team. Efforts were made to ensure diverse expertise, sex, and geographical representation. The group included individuals from 7 countries (countries included: Thailand, Zimbabwe, Kenya, South Africa, United Kingdom, United States, and Switzerland) and 4 continents, with expertise spanning from biomedical research to implementation science and qualitative research; representing research institutions, policy makers, and community. During phase 1, this group provided input on the scope of the project, defined the context in which the research priorities should be set, and agreed on the scoring criteria to be used for prioritization in phase 4.

Considering the aim to end the AIDS epidemic by 2030,^3^ the time frame for research priorities identified by the project was defined as present up to 2030. The process covered HIV testing, treatment, and service delivery. The populations of interest were children (0 < 10 years of age) and adolescents (10–19 years of age) living with HIV and those who require testing (this included HIV-exposed uninfected infants). HIV prevention was excluded because current efforts are underway to prioritize research in this area. (https://www.fic.nih.gov/About/center-global-health-studies/Pages/adolescent-hiv-prevention-treatment-implementation-science-alliance.aspx). The exercise was open to all types of research methodologies (eg, quantitative and qualitative) in the different research domains (basic, clinical, and operational) to be answered by multiple types of study designs (eg, observational, randomized trials, modeling, etc.).

The outcomes of importance were predefined by the working group. When formulating research questions, respondents were asked to consider both individual outcomes, eg, mortality, morbidity, biological markers, psychosocial well-being etc., and program outcomes, eg, linkage to care, retention, feasibility, acceptability, cost etc.

The recommended CHNRI scoring criteria^6^ were adapted, in discussion with the expert working group, to fit this process (Table 1) and as a result, “deliverability” was redefined as “implementation.” Clarity of the research questions was addressed by the working group before survey 2 (see phase 4). The “4D framework” (“description,” “delivery,” “development,” and “discovery” research) proposed by CHNRI was used to systematically categorize the research ideas. Minor adjustments were made to tailor the domains to the context of HIV (Table 2).

**TABLE 1.**
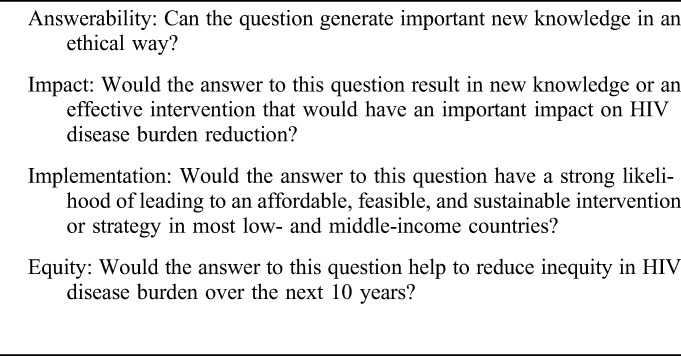
Scoring Criteria for Prioritization in Phase 4 of the Process

**TABLE 2.**
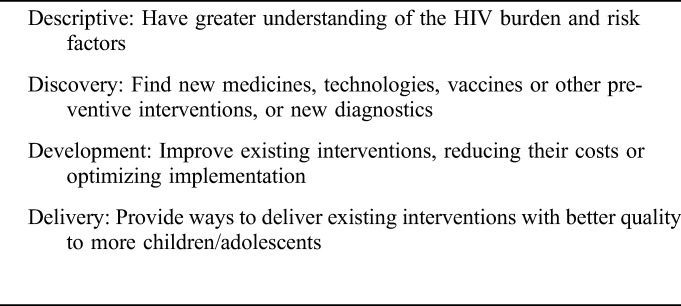
Research Question Domain Type

### Phase 2: Submission of Priority Research Questions

The first online survey, using Inquisium by Cvent, was shared using multiple dissemination pathways. The aim was to ensure inclusion of a broad range of stakeholders including researchers, program managers, clinicians, implementers, community partners, advocates, and young people, covering different epidemic and geographic settings. Using targeted snowballing, concentrated dissemination efforts were undertaken through (1) WHO country offices to ensure engagement of national program managers, (2) established community and youth networks, (3) postings on WHO and IAS websites and social media, and (4) the IAS and CIPHER member list serve of 3631 professionals working on pediatric and adolescent HIV. Respondents were encouraged to share the survey with their relevant networks.

Participants could submit up to 10 priority research questions across the 3 research areas, based on their knowledge and expertise for either one or both populations. Respondents were also asked to tag their question with the relevant research area and domain. The first online survey and associated communications were available in English, French, Spanish, and Portuguese.

### Phase 3: Thematic Content Analysis of Priority Research Questions Submitted

Non-English responses were back-translated. The data were cleaned by removing incomplete questions and those that did not meet the scope of the exercise (eg, relating to prevention, adult populations etc.). The research questions were then screened and sorted into population groups and research area of testing, treatment, and service delivery.

Qualitative thematic content analysis was used to analyze the research questions submitted.^14,15^ After the data were cleaned and sorted, recurring patterns were identified and categorized into themes and subthemes by A.A. and C.I. with the additional review and technical support from M.P. and M.V. The themes and subthemes identified were compared, harmonized, and refined across both populations. Where less than 3 questions were identified for any given theme, these were excluded from subsequent steps. Based on the thematic analysis, the research questions that addressed similar concepts were merged and formulated to find optimal balance between the breadth and details of the research questions submitted. This helped to minimize the overall number of questions to be scored in phase 4. The final collated lists of research questions were reviewed by the expert working group for clarity, consistency, and structure.

### Phase 4: Scoring of Research Questions

The collated merged research questions were uploaded onto SurveyMonkey Inc (Location: San Mateo, CA; Main Website: www.surveymonkey.com) to form the second survey. SurveyMonkey allowed the order of questions to be randomized per respondent, thus reducing preferential bias due to scoring fatigue. Respondents of survey 1 were invited to score a minimum of 2 research areas that related most to their expertise for one or both populations using predetermined criteria (Table 1). The CHNRI working group indicates that collective opinion reaches saturation at around 45–55 experts; therefore, survey 2 was closed once at least 45 individuals had responded per research area.^16^ Survey 2 was only available in English as the non-English response rate of survey 1 was below the predefined threshold of 10%.

**FIGURE 1.**
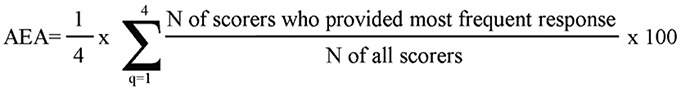
Formula used to calculate the Average Expert Agreement (AEA) score.

Based on other CHNRI processes, participants scored the answers to each criterion, which were then converted to a score (“yes” scored 100, “possibly” scored 50, and “no” scored 0). Participants could leave a response blank if they did not feel sufficiently informed to make a judgment. Blank responses were left out of the calculation of both numerator and denominator.^8^ Rankings were based on the total Research Priority Score (RPS) according to the formula:

RPS = [(answerability × 0.86) + (impact × 1.56) + (implementation × 0.77) + (equity × 0.81)]/4. The RPS was based on the mean suggested weights according to published guidelines from CHNRI stakeholders^8,17^ but rescaled to 4 (instead of 5) criteria with a maximum total numerator of 4, then adjusted to a 100-point scale. In addition, the Average Expert Agreement (AEA) scores were calculated, which represent the average proportion of scorers who agreed on responses for each of the 4 criteria asked (Fig. 1).

### Phase 5: Identification of Top Five Thematic Priorities per Research Areas

This final phase was an addition to the established CHNRI method described in phases 1–4 above. The working group members, with additional experts chosen to increase diversity (geographical and age) and strengthen some key areas of expertise, were charged with identifying 5 priority themes among the top 10 ranked questions in each research area. This involved (1) reviewing the ranked lists in the context of existing policy, systematic reviews, recently published research, and planned and ongoing research, and (2) participation in a webinar with structured facilitated discussion to reach consensus.

To inform this phase, an Excel template was generated per population, and divided into testing, treatment, and service delivery. Each area was organized by (1) the CHNRI results of ranked research questions, (2) the relevant WHO HIV recommendations^18,19^ and related systematic reviews, and (3) corresponding ongoing and planned studies. A broad PubMed search identified publications from June 2015, when latest systematic reviews were conducted for WHO HIV guidelines, to February 2017. Broad search terms relating to the 2 populations and HIV were used. These were coded thematically according to research areas and themes and provided as an additional resource. Ongoing and planned research was informed by the participants of survey 1 who, in addition to submitting the research questions, provided information on research projects being undertaking or planned. All types of study designs were accepted. This was strengthened with results from additional searches in clinicaltrials.gov and sorted to match the top 10 ranked questions per research area.

Two virtual meetings per population were held with the expert group to review the templates and make decisions on the top 5 research themes. The webinar was structured into 4 consensuses building steps^20^ (1) gathering initial feedback and clarifications, (2) facilitated discussion, (3) defining emerging proposals and changes, and (4) testing for consensus on the final top 5 themes. All participants had the opportunity to review and provide final agreement on the research themes.

### Ethical Considerations

No identifiable information was linked to respondent submissions and no IP addresses were recorded during the process. Contact information was used to forward respondents the second survey and to send out reminders. All participants could exit both surveys at any time.

## RESULTS

A total of 375 participants submitted 1735 priority research questions (an average of 4.6 questions per person) between September and October of 2016, with 58% submitting questions on both adolescent and pediatric HIV. Submissions were received from over 70 countries from all WHO regions, with most from the African region. Of the participants who submitted research questions, 55% (n = 206) identified themselves as researchers. The characteristics of the respondents for surveys 1 and 2 show similar composition (Table 3).

**TABLE 3.**
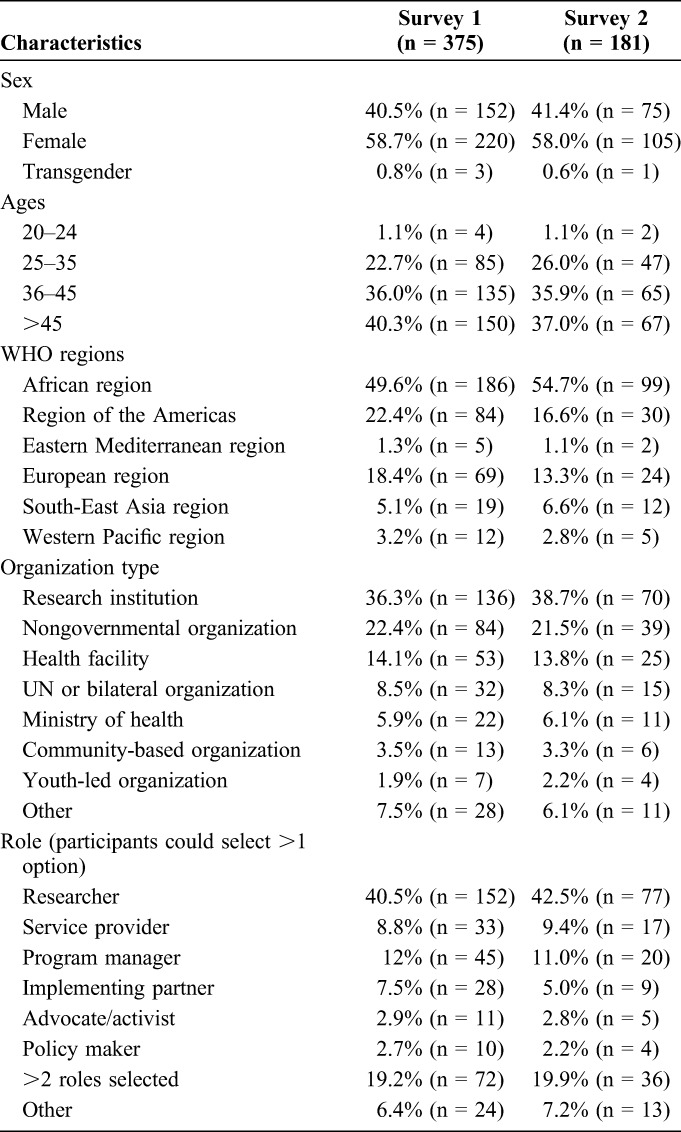
Characteristics of Respondents to Surveys 1 and 2

**FIGURE 2.**
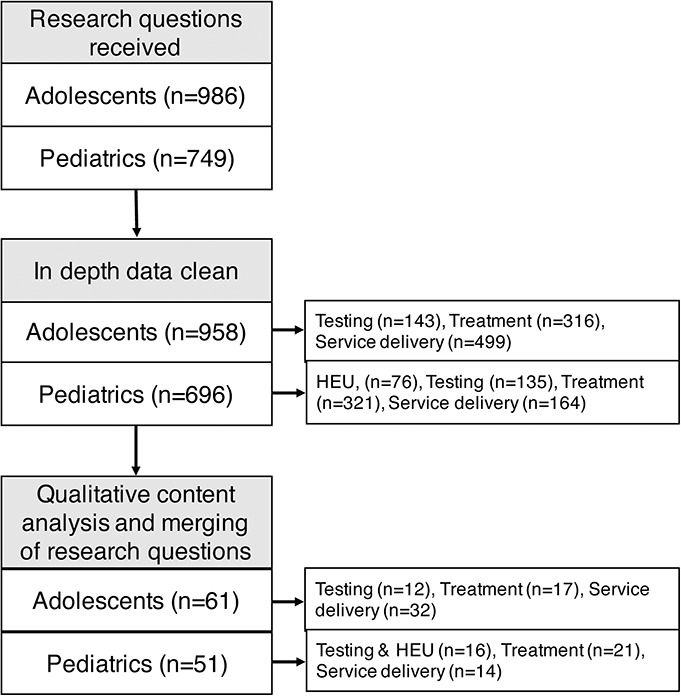
Flow diagram of data analysis process.HEU, HIV Exposed Uninfected.

After analysis, the final collated lists included 51 research questions and 61 research questions related to pediatric HIV and adolescent HIV, respectively (Fig. 2). Of the participants of survey 1 who were invited to participate in survey 2, 48% (n = 181) scored the collated lists of research questions.

Efforts to gather information on ongoing and planned studies resulted in the identification of the following: 37 testing, 57 treatment, and 19 for service delivery studies for pediatric HIV and 27 testing, 49 treatment, and 30 service delivery studies for adolescent HIV, mapped against the top 10 research questions per area. The PubMed scoping resulted in 249 and 65 published studies corresponding to the top 10 research questions for pediatric HIV and adolescent HIV, respectively. The final top themes identified and the full CHNRI ranked lists of research questions are discussed in separate publications.^11,12^

## DISCUSSION

The CHNRI methodology proved to be a highly comprehensive process that ensured a systematic, transparent, open, and inclusive approach to setting research priorities in pediatric and adolescent HIV. The novel adaptation of the CHNRI process for this exercise involved developing 5 top-priority thematic areas from the top 10 ranked research questions, within the context of the current research landscape. This process was also distinctive for 3 reasons. First, it addressed 2 populations in parallel, both children and adolescents. As we see children are surviving into adolescence there is an increased overlap in expertise, reflected by 58% of respondents who submitted questions for both populations. Second, the collaboration of WHO and CIPHER in leading the process, joined by their mandates to guide research, allowed for extensive reach to an existing group of engaged experts and IAS members involved in the HIV response. This was further facilitated by the already strongly connected and collaborative nature of HIV pediatric and adolescent networks and support of the expert working group. Third, it covered the HIV cascade of care across testing, treatment, and service delivery.

### Limitations and Strengths

#### The Top Five Thematic Areas

The additional phase to identify top themes served several functions: to ensure the relevance of the product, to provide a clear digestible outcome for users, to acknowledge the context of current research efforts, and to both facilitate communication and action. This phase was also in response to rapidly evolving research, especially because over the past 2 years, there has been an increased focus across several international agendas and initiatives^12,13,21,22^ recognizing the unique needs and services required for these populations. The strength of this additional step was that it outlined the existing body of evidence, highlighted key evidence gaps, and provided up-to-date knowledge of the research landscape, complementing that of the expert working group. Themes were used to separate this final step from the ranked lists of research questions identified. Although one previous study conducted content analysis to identify themes across 2 separate CHNRI exercises,^23^ to the best of our knowledge, this is the first example of identifying prioritized thematic areas based on the current evidence base. It is important to note that it was neither possible to comment on the quality of the ongoing and planned research nor the recently published literature identified, nor was it feasible to account for studies recently completed but not yet published. With the provision of the extensive mapping and the systematically formatted online consultations, it was the role of the expert resource group to highlight additional evidence gaps and reach consensus on the thematic outcomes. Although the themes communicate clearly the priority areas, the full ranked lists of research questions identified through the CHNRI method are also available and should be considered for future research.^11,12^

#### Stakeholder Involvement

It was important that there was wide-ranging multisectorial and multidisciplinary stakeholder inclusion to ensure that priorities reflected the range of priority areas across the HIV cascade and to support ownership of the exercise.^24^ Extensive outreach was performed, including snowballing, with the disadvantage that for the first survey, a respondent rate could not be calculated. Nonresponse rate bias therefore cannot be disregarded for survey 1. However, overall, there was broad geographical representativeness, including a majority from the African region, which is reflective of the geographic center of the HIV epidemic. Despite active efforts, engagement of younger age groups, ministries of health, and community-based organizations, who are an important part of the HIV response, was lower and future methods of how best to increase their participation will need to be considered.

#### Analysis of Questions Submitted

Efforts were made to ensure that the length of the research lists for survey 2 were manageable to reduce scoring fatigue and promote response rate, while at the same time addressing both the detail and breadth of the questions submitted by stakeholders. It cannot be discounted that this approach may have introduced bias^5^ from those compiling the final lists. However, the focused health topic area probably allowed for more extensive merging of complementing research ideas compared with other CHNRI processes that often address multiple health issues. Language was kept as consistent as possible with the submitted questions and, where appropriate, in line with language used by WHO HIV guidelines. Because the number of questions submitted was around 5 questions per person, future CHNRI efforts could propose that 5 or fewer questions be submitted to reduce time and resources required for analysis phase.

### Impact of the Process

Assessing the impact of research prioritization efforts is important, although challenging. One group assessed the impact of their work by quantifying the volume of publications since their prioritization exercise. Despite performing a comprehensive review, they concluded that the outcomes could not clearly be attributable to the priorities set.^25^ Future efforts could consider repeating the policy and research mapping exercise in 5–10 years and comparing it with the results of the mapping performed for this process. This may give an indication of the research volume in the priority areas identified. CHNRI has also suggested conducting interviews with key stakeholders to evaluate their uptake and implementation of the research agenda.^5^ Given the wide stakeholder engagement in this process and to address the question of attributability, the impact of this process will be in part measured through a survey of the different stakeholder groups on their implementation of the agendas, including funders and researchers. Through this process, CHNRI proved to be an inclusive, clear, transparent, and systematic method for setting research priorities, thus improving on previous consultative methods used by the WHO Department of HIV and IAS.

## CONCLUSIONS

To date, this is the largest example of the CHNRI priority setting exercise, in terms of stakeholders reached, in pediatric and adolescent HIV, and the first to incorporate top thematic areas based on current evidence. Led by global organizations and experts working in HIV, the resulting research agendas were developed through extensive inclusion of global stakeholders. The overall collective knowledge of the 375 individuals who participated is likely to be more reflective of the priorities in the field than previous research prioritization efforts performed. The additional step of reviewing this collective knowledge in the context of an up-to-date research landscape and highlighting thematic priorities further focuses the research agendas on what is really needed. The research agendas are intended to be adopted by funders and researchers to increase funding available and focus the research where evidence is most needed. A call to action has been launched by CIPHER and WHO urging all stakeholders to support implementation of the agendas through engagement in their respective roles, and highlighting the importance of involving people living with HIV in all aspects of its implementation in a meaningful and responsible way. The impact it will have on improving outcomes for these populations will depend on strong stakeholder leadership, optimized research efforts, and political and financial commitment.

## References

[1] UNAIDS. Global AIDS Update 2016. Geneva, Switzerland: UNAIDS; 2016 Available at: http://www.unaids.org/sites/default/files/media_asset/global-AIDS-update-2016_en.pdf. Accessed August 14, 2017.

[2] Penazzato M Amzel A Abrams EJ Pediatric treatment scale-up: the unfinished agenda of the global plan. J Acquir Immune Defic Syndr. 2017;1:S59–S65. 10.1097/QAI.0000000000001333 28398998

[3] UNAIDS. On the Fast-Track to End AIDS. 2016–2021 Strategy. Geneva, Switzerland: UNAIDS; 2015.

[4] Yoshida S Approaches, tools and methods used for setting priorities in health research in the 21st century. J Glob Health. 2016;6:010507. 26401271 10.7189/jogh.06.010507 PMC4576459

[5] Rudan I Yoshida S Chan KY Setting health research priorities using the CHNRI method: VII. A review of the first 50 applications of the CHNRI method. J Glob Health. 2017;7:011004. 28685049 10.7189/jogh.07.011004 PMC5481891

[6] Rudan I Gibson JL Ameratunga S Setting priorities in global child health research investments: guidelines for implementation of the CHNRI method. Croat Med J. 2008;49:720–733. 19090596 10.3325/cmj.2008.49.720 PMC2621022

[7] Hindin MJ Sigurdson Christiansen C Ferguson J Setting research priorities for adolescent sexual and reproductive health in low and middle income countries. Bull World Health Organ. 2013;91:10–18. 23397346 10.2471/BLT.12.107565 PMC3537249

[8] Nagata JM Ferguson BJ Ross DA Research priorities for eight areas of adolescent health in low- and middle-income countries. J Adolesc Health. 2016;59:50–60. 27235375 10.1016/j.jadohealth.2016.03.016 PMC5357763

[9] Rollins N Chanza H Chimbwandira F Prioritizing the PMTCT implementation research agenda in 3 African countries: INtegrating and scaling up PMTCT through implementation REsearch (INSPIRE). J Acquir Immune Defic Syndr. 2014;67:S108–S113. 25310115 10.1097/QAI.0000000000000358

[10] UNAIDS and The Henry JKaiser Family Foundation. Financing the Response to HIV in Low- and Middle-Income Countries in 2016. Geneva, Switzerland: UNAIDS and The Henry J Kaiser Family Foundation; 2017 Available at: http://www.unaids.org/en/resources/documents/2017/20170721_Kaiser_donor_funding_HIV_LMIC_2016. Accessed July 26, 2017.

[11] Penazzato M Vicari M Irvine C A global research agenda for paediatric HIV. J Acquir Immune Defic Syndr. 2018;78(suppl 1):S10–S15. 29994914 10.1097/QAI.0000000000001743 PMC6075892

[12] Armstrong A Nagata J Baggely R A global research agenda for adolescent HIV. J Acquir Immune Defic Syndr. 2018;78(suppl 1):S16–S21. 29994915 10.1097/QAI.0000000000001744 PMC6075888

[13] Rudan I Setting health research priorities using the CHNRI method: IV. Key conceptual advances. J Glob Health. 2016;6:010501. 27418959 10.7189/jogh-06-010501 PMC4938380

[14] Javadi M Zarea K Understanding thematic analysis and its pitfall. J Clie Care. 2016;1:34–40.

[15] Elo S Kääriäinen M Kanste O Qualitative content analysis: a focus on trustworthiness. SAGE Open. 2014;4:1–10.

[16] Yoshida S Rudan I Cousens S Setting health research priorities using the CHNRI method: VI. Quantitative properties of human collective opinion. J Glob Health. 2016;6:010503. 27350874 10.7189/jogh.06.010503 PMC4920008

[17] Kapiriri L Tomlinson M Chopra M Setting priorities in global child health research investments: addressing values of stakeholders. Croat Med J. 2007;48:618–627. 17948948 PMC2213572

[18] World Health Organization. Consolidated Guidelines on the Use of Antiretroviral Drugs for Treating and Preventing HIV Infection: Recommendations for a Public Health Approach. Geneva, Switzerland: World Health Organization; 2016 Available at: https://www.ncbi.nlm.nih.gov/books/NBK374294/. Accessed August 28, 2017. 27466667

[19] World Health Organization. Consolidated Guidelines on HIV Testing Services. Geneva, Switzerland: World Health Organization; 2015 Available at: http://www.who.int/hiv/pub/guidelines/hiv-testing-services/en/. Accessed August 28, 2017.

[20] Seeds for Change. A Consensus Handbook, Cooperative Decision Making for Activists, Coops and Communities. United Kingdom: Seeds for Change, Lancaster Co-operative Ltd; 2013 Available at: https://www.seedsforchange.org.uk/consensus. Accessed August 28, 2017.

[21] UNICEF. ALL in to #EndAdolescentAIDS. Available at: https://childrenandaids.org/all-in-to-end-adolescent-AIDS. Accessed August 28, 2017.

[22] The United States President's Emergency Plan for AIDS Relief. Accelerating Children's HIV/AIDS treatment (ACT). Available at: https://www.pepfar.gov/priorities/children/index.htm. Accessed August 28, 2017.

[23] Nagata JM Hathi S Ferguson BJ Research priorities for adolescent health in low- and middle-income countries: a mixed-methods synthesis of two separate exercises. J Glob Health. 2018;8:010501. 29497507 10.7189/jogh.08.010501 PMC5825976

[24] Viergever RF Olifson S Ghaffar A A checklist for health research priority setting: nine common themes of good practice. Health Res Policy Syst. 2010;8:36. 21159163 10.1186/1478-4505-8-36 PMC3018439

[25] Odone A Matteelli A Chiesa V Assessing the impact of defining a global priority research agenda to address HIV-associated tuberculosis. Trop Med Int Health. 2016;21:1420–1427. 27576587 10.1111/tmi.12768

